# Identification and Characterization of *Short Crown Root 8*, a Temperature-Sensitive Mutant Associated with Crown Root Development in Rice

**DOI:** 10.3390/ijms22189868

**Published:** 2021-09-13

**Authors:** Peng Hu, Yi Wen, Yueying Wang, Hao Wu, Junge Wang, Kaixiong Wu, Bingze Chai, Lixin Zhu, Guangheng Zhang, Zhenyu Gao, Deyong Ren, Li Zhu, Longbiao Guo, Dali Zeng, Jing Xu, Song Yan, Qian Qian, Yuchun Rao, Jiang Hu

**Affiliations:** 1 State Key Laboratory of Rice Biology, China National Rice Research Institute, Hangzhou 310006, China; 15168331324@126.com (P.H.); Wenyi199492@hotmail.com (Y.W.); wyywangyueying@163.com (Y.W.); wuhaoge1987@163.com (H.W.); 15038793321@163.com (J.W.); 15932930488@163.com (K.W.); 13204660061@163.com (B.C.); zhulixin96@foxmail.com (L.Z.); zhangguangheng@126.com (G.Z.); zygao2000@hotmail.com (Z.G.); rendeyongsd@163.com (D.R.); zhuli05@caas.cn (L.Z.); guolongbiao@caas.cn (L.G.); dalizeng@126.com (D.Z.); xujing87@126.com (J.X.); qianqian188@hotmail.com (Q.Q.); 2 Agricultural Genomics Institute at Shenzhen, Chinese Academy of Agricultural Sciences, Shenzhen 518120, China; 3 Rice Research Institute of Shenyang Agricultural University/Key Laboratory of Northeast Rice Biology and Breeding, Ministry of Agriculture, Shenyang 110866, China; 4 Rice Research Institute, Jiangxi Academy of Agricultural Sciences, Nanchang 330200, China; yans11@163.com; 5 College of Chemistry and Life Sciences, Zhejiang Normal University, Jinhua 321004, China

**Keywords:** crown root, defense response, temperature-sensitive, *scr8*, rice

## Abstract

Crown roots are essential for plants to obtain water and nutrients, perceive environmental changes, and synthesize plant hormones. In this study, we identified and characterized *short crown root 8* (*scr8*), which exhibited a defective phenotype of crown root and vegetative development. Temperature treatment showed that *scr8* was sensitive to temperature and that the mutant phenotypes were rescued when grown under low temperature condition (20 °C). Histological and EdU staining analysis showed that the crown root formation was hampered and that the root meristem activity was decreased in *scr8*. With map-based cloning strategy, the *SCR8* gene was fine-mapped to an interval of 126.4 kb on chromosome 8. Sequencing analysis revealed that the sequence variations were only found in *LOC_Os08g14850*, which encodes a CC-NBS-LRR protein. Expression and inoculation test analysis showed that the expression level of *LOC_Os08g14850* was significantly decreased under low temperature (20 °C) and that the resistance to *Xanthomonas oryzae pv. Oryzae* (*Xoo*) was enhanced in *scr8*. These results indicated that *LOC_Os08g14850* may be the candidate of *SCR8* and that its mutation activated the plant defense response, resulting in a crown root growth defect.

## 1. Introduction

Plant roots determine the absorption of water and nutrients and fix and support the aboveground parts, which directly affect plant growth and development [1]. Plant root systems are divided into two main types: fibrous root systems and taproot systems. Dicotyledons possess taproot systems, which include the main root and lateral roots, while monocotyledons have fibrous root systems, including primary roots, crown roots (also called adventitious roots), and lateral roots. Rice is a monocotyledonous plant, and its crown roots mainly undergo three stages of development: initiation, emergence, and elongation, in which the abnormal development will directly affect the morphology of roots [2,3]. 

Root growth and development are derived from stem cells, which divide into root apical meristem (RAM) to enter the elongation/differentiation zone to initiate cell differentiation [4]. To ensure root meristem maintenance, the cell division and differentiation must be coordinated, and disturbance of the balance will affect the RAM activity, resulting in abnormal development of root length and size [4,5,6]. Several regulatory pathways have been reported to affect crown root development, in which the generation and differentiation of RAM plays an important role. WOX11 encodes a WUSCHEL (WUS)-related homeobox protein that regulates crown root meristem cell proliferation by recruiting the ADA2-GCN5 histone acetyltransferase module to activate downstream target genes [7]. ROOT MERISTEM GROWTH FACTOR 1 (RGF1) is a critical peptide hormone, and RGF1-RGI1 determines primary root growth and development by regulating the expression of PLT1/PLT2 via the YDA-MKK4/MKK5-MPK3/MPK6 signaling cascade [8,9]. The microRNAs (miRNAs) are endogenous small non-coding RNAs that mediate crown root development. *LOWER CROWN ROOT NUMBER 1* (*LCRN1*) encodes SQUAMOSA PROMOTER BINDING PROTEIN-LIKE 3(SPL3), which regulates crown root development through the OsmiR156-OsSPL3/OsSPL12 pathway [10]. Xpo1 domain protein of CROWN ROOT DEFECT 1 (CRD1) is essential for maintaining normal miRNA levels, and its mutation leads to the disruption of the miR156 regulatory pathway and significant inhibition of crown root development [11]. In addition, temperature is also an important external environmental factor affecting crown root development. *BRASSINOSTEROID INSENSITIVE 1* (*BRI1*) mediates root elongation by downregulating brassinosteroid signaling at high temperatures [12]. Loss-of-function of *ADAPTATION TO ENVIRONMENTAL TEMPERATURE 1* (*AET1*) results in a short crown root and insensitivity to auxin in high temperatures [13]. Moreover, the root growth defects caused by hybrid weakness can also be recovered by low-temperature treatment [14].

Several factors affecting the balance between cell division and cell differentiation are involved in regulating root meristem activity. The complex network of interactions of hormonal pathways plays a key role in root meristem activity regulation. Plant endogenous hormones, such as auxin, cytokinin (CK), gibberellin (GA), salicylic acid (SA), and jasmine acid (JA), are critical factors involved in root development [15]. Among them, auxin promotes root growth and development, and plant treatment with auxin induces lateral root and adventitious root formation [16,17]. In *Arabidopsis thaliana*, *MUSTACHES* (*MUS*) and *MUSTACHES-LIKE* (*MUL*) were regulated by *AUXIN RESPONSE FACTOR7* (*ARF7*) and *ARF19*, and the mutations of *MUS* and *MUL* inhibited lateral root growth [18,19]. Moreover, the *PR-1* HOMOLOG gene (*PRH*) was found to regulate lateral root formation through the ARF7/LBD pathway [20,21]. In rice, *CROWN ROOTLESS 1* (*CRL1*)/ *ADVENTITIOUS ROOTLESS 1* (*ARL1*) encodes a LOB-domain transcription factor and regulates crown root formation by positively regulating the expression of meristem- and cell proliferation-related gene [22,23,24]. In contrast with auxin, cytokinin plays an inhibitory effect on root growth. *WOX11* interacts with *AP2/ETHYLENE-RESPONSIVE FACTOR* (*ERF3*) to regulate the expression of *CYTOKININ TYPE-A RESPONSIVE REGULATOR* (*RR2*) during crown root formation and development [2,25]. *ROOT ENHANCER 1* (*REN1*)/*CYTOKININ OXIDASE/DEHYDROGENASE 4* (*CKX4*) affects crown root formation by integrating the cytokinin and auxin pathways [26]. Recent studies have found that the E3 ubiquitin ligase complex *APC/CTE* is activated by high gibberellins levels and inhibits root meristem growth by mediating SHORTROOT 1 (OsSHR1) degradation [27]. In addition, SA and JA regulate crown root growth. The short root phenotype of *ABNORMAL INFLORESCENCE MERISTEM1* (*AIM1*) mutant is rescued by SA treatment in rice [6], and JA participates in inducing the activation and regeneration of root stem cells by regulating the RBR-SCR network and the stress response protein ERF115 in *Arabidopsis thaliana* [28].

To date, few genes have been reported to be involved in crown root development. Therefore, the identification of new crown root mutants can help further cloning of related genes and enriching its molecular regulatory network. In this study, we identified a novel short crown root mutant that displayed reduced root growth phenotype. The *scr8* is temperature sensitive and exhibits short crown root at high temperature (32 °C) and presents normal crown root at low temperature (20 °C). Map-based cloning revealed that *LOC_Os08g14850* may be the candidate gene that encodes a CC-NB-LRR-type protein involved in the plant defense response. We demonstrated that the mutation of *SCR8* disturbed the balance between plant growth and defense response, resulting in the crown root growth inhibition.

## 2. Results

### 2.1. Identification of Scr8 Mutant 

**Figure 1:**
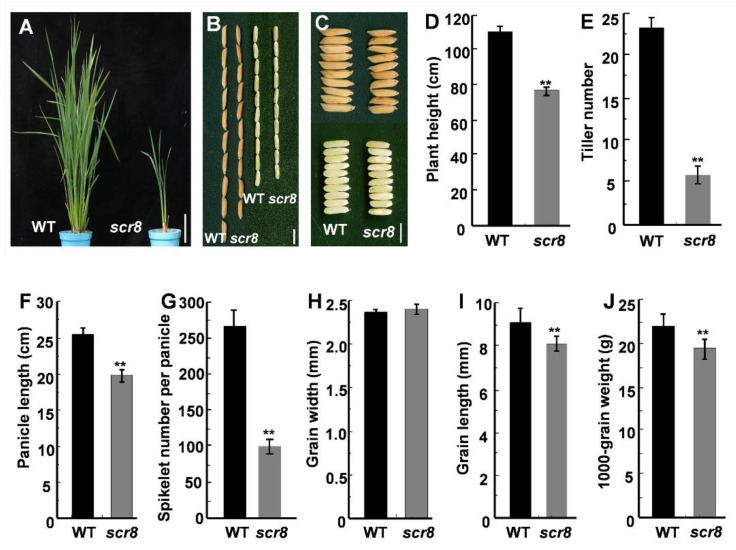
Characterization of WT and *scr8* mutant. (**A**) Plant architecture of WT and *scr8* mutant plants at the booting stage. Scale bars, 10 cm. (**B**,**C**) Mature paddy rice grains and brown rice grains from WT and *scr8*. Scale bars, 0.5 cm. (**D**–**J**) Comparisons between WT and *scr8* for average plant height (n = 15 plants) (**D**), average tiller number (n = 15 plants) (**E**), average panicle length (n = 15 plants) (**F**), average spikelet number per panicle (n = 15 plants) (**G**), average grain width (n = 15 plants) (**H**), average grain length (n = 15 plants) (**I**), 1000-grain weight (n = 15 plants) (**J**). Error bars represent standard deviation (SD). ** Significant difference at *p* < 0.01 compared with the WT by Student’s *t*-test.

**Figure 2:**
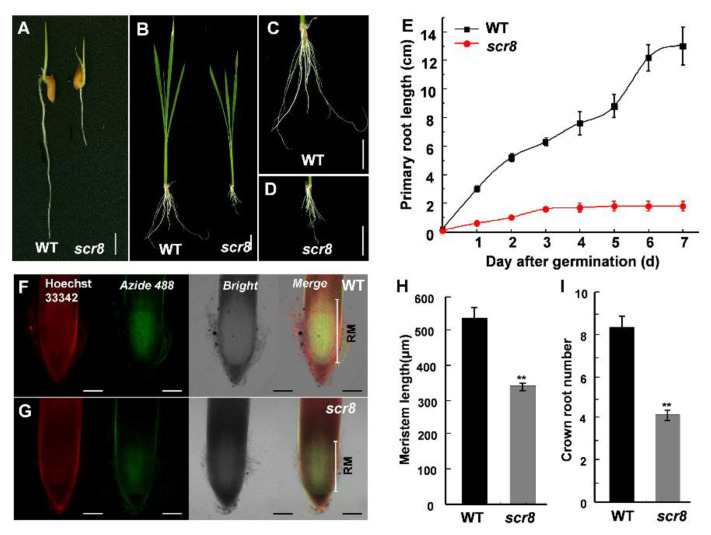
The meristem activity of WT and *scr8*. (**A**) Root phenotype of 3-day-old WT and *scr8*. Scale bars, 1 cm. (**B**–**D**) Root phenotype of 30-day-old WT and *scr8*. Scale bars, 1 cm. (**E**) Primary root length of WT and *scr8* after germination (n = 10). (**F**) and (**G**) EdU staining analysis of 3-day-old WT and *scr8*. Scale bars, 100 μm. (**H**) Meristem length of 3-day-old WT and *scr8* (n = 3). (**I**) Crown root number of 7-day-old WT and *scr8* (n = 10). Error bars represent SD. ** Significant difference at *p* < 0.01 compared with the WT by Student’s *t*-test.

The *scr8* mutant was obtained from a natural variation of *indica* variety R1206. Phenotypic observation showed that the mutant exhibited reduced growth, including shortened plant height, panicle length, and reduced tiller number (Figure 1A,D–F). Further phenotypic measurements showed that grain length, 1000-grain weight, and spikelet number of panicles were also significantly reduced, but there was no change in grain width compared with wildtype (WT) (Figure 1B,C,H–J). In addition, the root development of *scr8* was significantly inhibited and the plants easily perished in the field (Figure 2A–D). These results show that the growth and development of *scr8* was seriously impaired, which eventually led to a reduction in biomass. 

### 2.2. Reduced Root Meristem Activity of Scr8

After germination, *scr8* initially showed a phenotype of impaired shoot and root growth, especially crown root (Figure 2A–E). Moreover, the elongation of crown root ceased on third day, and the root number was also significantly reduced in *scr8* compared with the WT (Figure 2E,I). To detect the differences in root meristem activity, EdU (5-ethynyl-29-deoxyuridine), a thymidine analog, was used to detect cell proliferation. The results show that the amount of EdU labeling was significantly reduced in the mutant compared with the WT (Figure 2F–H), indicating root meristem activity was decreased in *scr8*. 

### 2.3. Anatomic Analysis of Scr8

**Figure 3:**
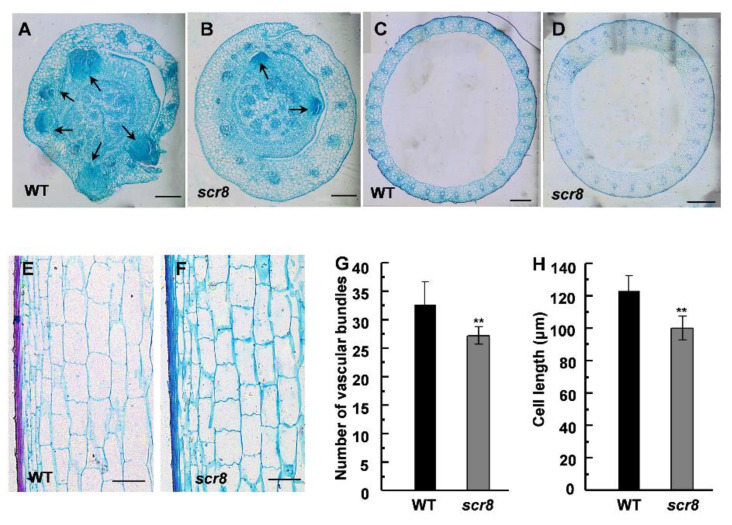
Histological characterization of basal nodes and culms. (**A**,**B**) Cross sections of basal nodes from WT and *scr8* mutant plants. Arrow represents crown root primordia. Scale bars, 200 μm. (**C**,**D**) Cross sections of culms from WT and *scr8* mutant plants. Scale bars, 200 μm. (**E**,**F**) Longitudinal sections of culms from WT and *scr8* mutant plants. Scale bars, 100 μm. (**G**) Average number of vascular bundles of culms in WT and *scr8.* (**H**) Average cell length of culms in WT and *scr8.* Error bars represent SD. ** Significant difference at *p* < 0.01 compared with the WT by Student’s *t*-test.

To further understand the *scr8* phenotype, the histological characteristics of the culms and basal nodes were observed by paraffin section. Due to crown root growth initiation in the tissue of root basal node, we carried out the microscopic observation. The result indicates that crown root formation was severely hampered and that the number of crown root primordia were significantly reduced (Figure 3A,B), which suggests that the defected crown root growth of *scr8* was mainly due to decreased meristem activity and fewer root primordia. Compared with the wildtype, the vascular numbers and cell length of culms were significantly reduced, which is consistent with the inhibited growth phenotype of *scr8* (Figure 3C–H).

### 2.4. Temperature Affected Scr8 Root Growth

**Figure 4:**
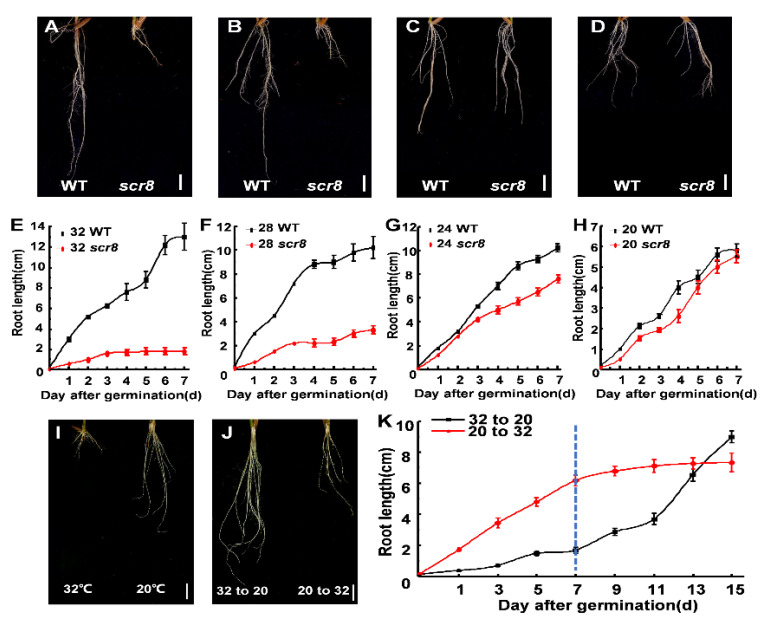
Different temperature treatment and temperature shift experiment. (**A**–**D**) Morphology of WT and *scr8* under various temperature conditions: 32 °C treatment (**A**), 28 °C treatment (**B**), 24 °C treatment (**C**), 20 °C treatment (**D**). Scale bars, 1 cm. (**E**–**H**) Time-course measurements of root length in WT and *scr8* mutant plants: 32 °C treatment (**E**), 28 °C treatment (**F**), 24 °C treatment (**G**), 20 °C treatment (**H**). (**I**,**J**) temperature shift experiment of *scr8.* Root phenotype of *scr8* grown at 32 °C and 20 °C (**I**), Root phenotype of *scr8* after temperature shift (**J**). Scale bars, 1 cm. “32 to 20” and “20 to 32” indicate that the temperature was changed from 32 °C to 20 °C or from 20 °C to 32 °C at 7 days after germination. (**K**) Time course analysis of root length of in response to temperature-shift treatment.

Temperature is a major abiotic stress in plant growth and development, including germination, growth, flowering, and disease resistance [13,14]. In this study, *scr8* was sensitive to high temperatures and displayed severe root growth defects above 28 °C (Figure 4A,B,E,F). However, at temperatures below 24 °C, the root development of *scr8* was normal, and the root lengths were the same as those of the wildtype (Figure 4C,D,G,H). In addition, the *scr8* growth defects were reversed by low-temperature treatment. We shifted the temperature from 32 °C to 20 °C, and root growth of *scr8* increased significantly. Conversely, *scr8* growth was inhibited when the temperature was increased from 20 °C to 32 °C (Figure 4I–K).

### 2.5. Scr8 Showed Increased SA and JA Content 

**Figure 5:**
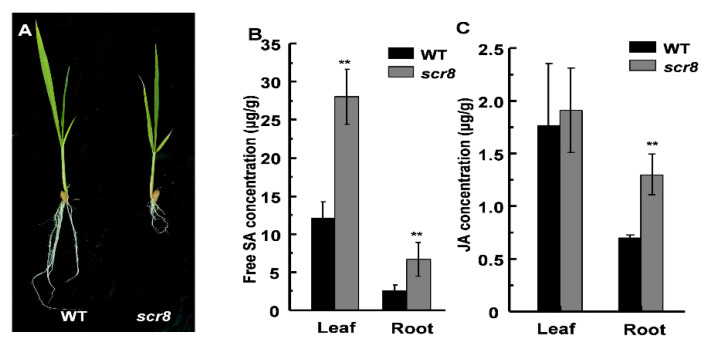
The contents of SA and JA in WT and *scr8.* (**A**) The phenotype of WT and *scr8* in seedling. Scale bar, 1cm. (**B**) Contents of free SA in WT and *scr8* leaves and roots (n = 3). (**C**) Contents of JA in WT and *scr8* leaf and roots (n = 3). Error bars represent SD. ** Significant difference at *p* < 0.01 compared with the WT by Student’s *t*-test.

Hormones play an important role in regulating plant growth and development, and previous studies have shown that the metabolism of SA and JA is a key factor in regulating plant root growth [6,29]. To clarify whether the *scr8* root phenotype is related to SA or JA, we measured the free SA and JA concentrations at the seedling stage and found higher accumulations of SA and JA in *scr8* compared with the wildtype (Figure 5B,C). Among them, the SA content in both *scr8* shoots and roots was significantly higher than WT, but the content of JA was only higher in roots. In fact, the mutant phenotype is more apparent in roots than shoots, which is consistent with the variation in JA content (Figure 5A–C). Therefore, we speculated that JA may be the main hormone affecting the development of *scr8*.

### 2.6. Fine Mapping of Scr8 and Candidate Gene Analysis 

**Figure 6:**
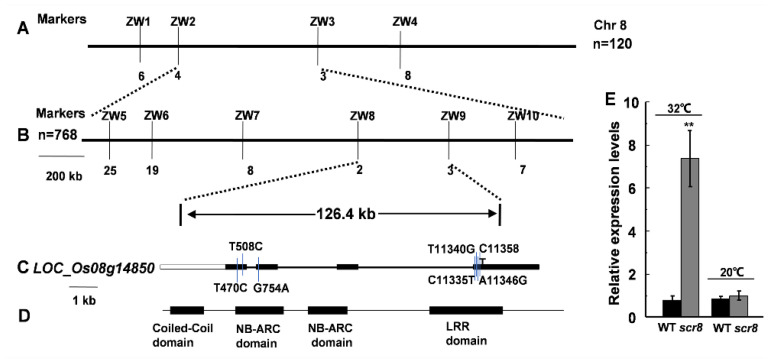
Fine mapping of the *SCR8* gene. (**A**,**B**) Fine mapping of the *SCR8* gene. (**C**,**D**) Structure analysis of SCR8. (**E**) Relative expression level of *LOC_Os08g14850* grown at 32 °C and 20 °C. Error bars represent SD. ** Significant difference at *p* < 0.01 compared with the WT by Student’s *t*-test.

To identify the mutant gene, an F_2_ population was developed by crossing *scr8* with *japonica* variety WYG7, and the segregation of wildtype and mutant phenotypes displayed a ratio of 3:1 (74 mutant-type: 255 WT-type, χ^2^ = 0.974 <χ^2^_0.05_ = 3.84), indicating that the mutant phenotype was controlled by a single recessive gene. *SCR8* was first mapped to chromosome 8, between molecular markers ZW2 and ZW3, and its location was then narrowed down to a 126.4 kb genomic region between the markers ZW8 and ZW9 using 768 homozygous mutant plants (Figure 6A,B). In this region, a total of 22 predicted opening reading frames (ORFs) were found according to the publicly available rice database, Rice Genome Annotation Project (http://rice.plantbiology.msu.edu/index.shtml accessed on 16 June 2021), in which a resistance protein gene of the NB-ARC domain, *LOC_Os08g14850*, was the most likely candidate (Figure 6C,D). The promoter and coding regions of the 22 genes were amplified and sequenced, and seven variations were found only in *LOC_Os08g14850*. We carried out qRT-PCR analysis, and the results indicate that the expression of *LOC_Os08g14850* was significantly increased in *scr8* when grown at 32 ℃, but there was no change at 20 ℃ (Figure 6E). Thus, we considered *LOC_Os08g14850* to be a candidate gene for *SCR8*.

### 2.7. Transcriptome Analysis of the Wildtype and Scr8 Mutant

**Figure 7:**
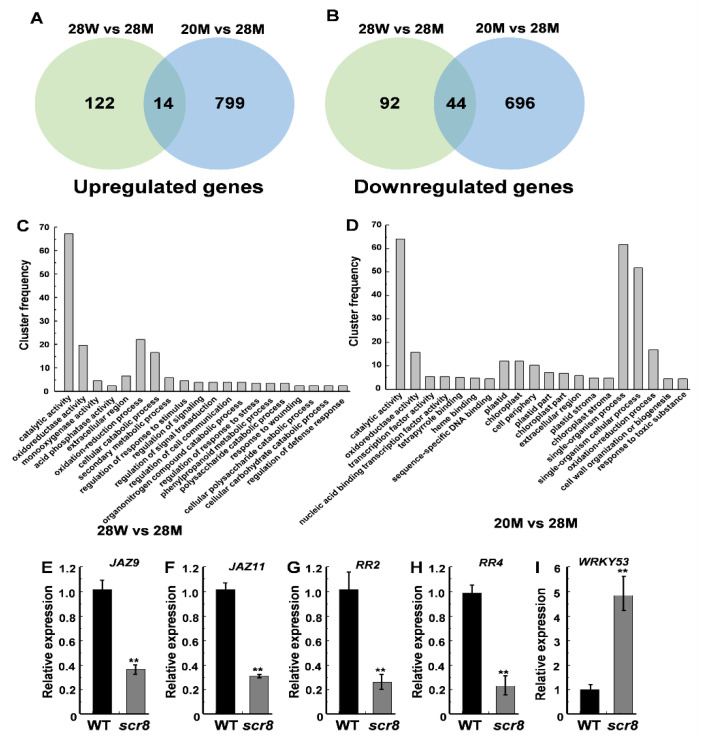
Transcriptome analysis of the WT and *scr8* under different temperature treatment. (**A**,**B**) Venn diagrams showing transcriptome changes in 7-day-old WT and *scr8* grown at 20 °C and 28 °C. The number of upregulated genes in *scr8* (**A**). The number of downregulated genes in *scr8* (**B**). (**C**) Top 20 significantly enriched GO terms compared between WT and *scr8* when grown under high temperature. (**D**) Top 20 significantly enriched GO terms in *scr8* after low temperature treatment. (**E**–**I**) Relative expression of *JAZ9, JAZ11, RR2, RR4*, and *WRKY53* genes in 7-day-old WT and *scr8*. Error bars represent SD. ** Significant difference at *p* < 0.01 compared with the WT by Student’s *t*-test.

To characterize the molecular regulatory network of *SCR8*, RNA-seq analysis was performed on plants grown at 20 °C and 28 °C. In total, 272 DEGs (differentially expressed genes, false discovery rate < 0.05 and induction fold > 1), including 136 upregulated genes and 136 downregulated genes between the WT and *scr8* were identified when grown at 28 °C, and 1553 DEGs (813 upregulated and 740 downregulated genes) between 20 °C and 28 °C were also found in *scr8* (Figure 7A,B). Gene ontology (GO) analysis classified most of the DEGs as being involved in catalytic activity, oxidoreductase activity, oxidation-reduction processes, and cellular catabolic processes in *scr8* compared with the WT when grown at 28 °C (Table S2). After low-temperature treatment (20 °C), the significantly enriched GO terms were catalytic activity, single-organism processes, single-organism cellular processes, and oxidoreductase activity (Figure 7C,D; Table S3). Among these DEGs, a number involved in cell cycle and hormone synthesis were identified, including *CycA1;1*, *CycD5;2*, *WRKY53*, *JAZ9*, *JAZ11*, *RR2*, and *RR4* (Tables S4 and S5). To evaluate the reliability of the RNA-seq data, we selected some upregulated and downregulated genes for validation by qRT-PCR, and the results were consistent (Figure 7E–I).

### 2.8. Scr8 Showed Enhanced Resistance to Xoo

**Figure 8:**
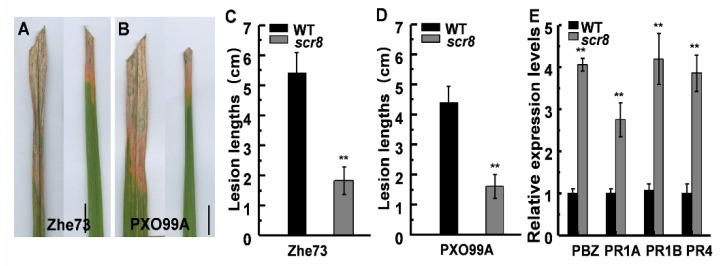
*scr8* induced pathogenesis-related genes and disease resistance. (**A**) Leaf phenotype of WT and *scr8* after inoculation with Zhe173. Scale bar, 1cm. (**B**) Leaf phenotype of WT and *scr8* after inoculation with PXO99A. Scale bar, 1cm. (**C**) Lesion length after inoculation Zhe173. (**D**) Lesion length after inoculation PXO99A. (**E**) Relative expression levels of pathogenesis-related genes in WT and *scr8* plants at heading stage. Error bars represent SD. ** Significant difference at *p* < 0.01 compared with the WT by Student’s *t*-test.

Since *LOC_Os08g14850* is predicted to be a resistance protein, and its expression was significantly enhanced in *scr8*, a bacterial blight inoculation test was carried out with *Xoo* strains Zhe173 and PXO99A at the heading stage. To quantify the pathogen responses, the lesion lengths were measured after 15 days, and the results show that the lesion lengths of *scr8* were significantly shorter than those of WT (Figure 8A–D). Moreover, the transcription level of pathogen-related genes (PRs) *OsPR1a*, *OsPR1b*, *OsPR4*, and *PBZ1* were detected, and the expression levels of these genes were also significantly higher in *scr8* (Figure 8E). 

## 3. Discussion

In past decades, several genes involved in regulating crown root growth and development have been identified and characterized in rice, including *CRL4/GNOM1*, *CRL5*, *SRT5*, and *WOX11* [2,25,29,30,31]. However, the molecular mechanisms of rice crown root development are still unclear. In this study, we isolated a new mutant *scr8* from *indica* R1206 involved in temperature response and hormone synthesis that affected the crown root growth and development. 

**Figure 9:**
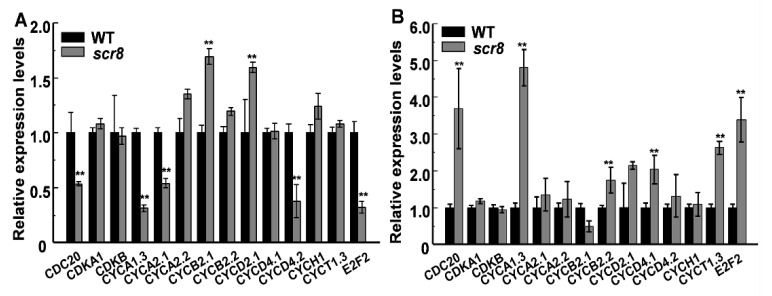
Expression analysis of genes related to cell cycle in WT and *scr8.* (**A**) Genes expression of WT and *scr8* grown at 32 ℃. (**B**) Genes expression of WT and *scr8* grown at 20 ℃. Error bars represent SD. ** Significant difference at *p* < 0.01 compared with the WT by Student’s *t*-test.

Root growth depends on cell proliferation and elongation, in which the former mainly occurs in the RAM, and root length is affected when meristem zone activity decreases, such as *GR2, DPR2*, and *CBLs* in plants [31,32,33,34,35,36,37]. In this study, the *scr8* mutant showed shorter primary roots and crown roots, decreased root meristem activity, and reduced meristem length (Figure 2F,G). In addition, the expression levels of cell-cycle-related genes *CDC2*, *CYCA1;3*, *CYCA2;1*, *CYCD4;2*, and *E2F2* were downregulated in *scr8* grown at 32 °C but significantly upregulated when grown at 20 °C (Figure 9A,B). Moreover, the RNA-seq data also exhibited significant changes in the expression of cell-cycle-related genes at different temperatures. These results indicate that cell division and elongation were inhibited and meristem activity was reduced in *scr8*, which led to the defect of crown root development in *scr8*.

In the long-term evolutionary process, plants have established complex and diverse resistance response systems to resist the invasion of pathogens and insects. However, the abnormality of plant defense response disturbs the balance between plant growth and resistance response, resulting in a variety of abnormal phenotypes, such as lesion mimic leaf, spotted leaf, shorter root, hybrid weakness, and so on [15,38,39,40,41,42,43]. Therefore, plants must coordinate the balance between immune response and growth in a constantly changing environment. The previous studies have shown that the proteins containing NB-ARC and LRR domains play key roles in the maintenance of the autoinhibited state, and the mutations of these domains activates plant defense responses [38,39,40,41]. NRTP1 encodes a typical coiled-coil nucleotide-binding leucine-rich repeat (CC-NB-LRR)-type protein, and its semi-dominant mutant showed defense response characteristics and exhibited shorter roots [39]. *Hwi1* comprises two loci—25L1 and 25L2—and both encode leucine-rich repeat receptor-like kinase (LRR–RLK), which is incompatible with *Hwi2* derived from wild rice (*Oryza rufipogon*). The hybrid weakness caused by the pyramiding of *Hwi1* and *Hwi2* activates an autoimmune response in the basal nodes of hybrids, interrupts root formation, and impairs shoot growth. [14]. Similarly, the candidate gene *LOC_Os08g14850* encodes a CC-NBS-LRR-type protein containing NB-ARC domain (Figure 6A–D). In addition, the expression of PR genes were significantly increased, and the inoculation experiment also showed that the resistance to pathogens was enhanced in *scr8* (Figure 8A,B,E). Thus, the interaction between the NB-ARC domain and LRR domain may be disrupted in *scr8*, resulting in auto-activation of the defense response. 

It is well known that ambient temperature significantly affects plant growth and development [15,44,45,46]. R or R-like proteins of NB-LRR type play a receptor-like function and participate in specific recognition of pathogens, which are likely the causal temperature-sensitive component in defense responses [41]. High temperature usually decreases plant resistance, but it occasionally activates resistance [15,41,47,48,49,50,51]. In this study, the *scr8* showed dwarf, short root phenotype, and the resistance to *Xoo* was enhanced more than wildtype under high temperature in filed. However, the *scr8* phenotype of crown root growth defect was rescued by low-temperature treatment (Figure 4A–D), and the expression of *LOC_Os08g14850* was significantly higher than WT at 32 ℃, but there was no change at 20 ℃ (Figure 6E). Therefore, the *scr8* may respond to high temperature signals and abnormally initiate defense responses. 

The reported crown root growth defect materials are usually related to plant hormone synthesis and metabolism disorders [2,6,52,53,54]. SA and JA are two well-known phytohormones involved in plant defense responses. Among them, SA plays an important role in maintaining the root meristem activity, and the decrease in SA content leads to a reduction in root meristem activity [6,55]. JA acts on the stem cells and tissue regeneration, and negatively regulates the adventitious root formation [16,56,57,58,59,60]. In *scr8*, the SA and JA were highly accumulated in the leaves and roots (Figure 5B,C), and the resistance to bacteria pathogen *Xoo* was also strikingly increased in leaves (Figure 8A,B), suggesting that plant defense responses were activated. RNA-seq and expression levels analysis also demonstrated these results, and EdU staining indicated that the root meristem activity had decreased in *scr8* (Figure 2F,G and Figure 7). Thus, we speculated that *SCR8* is required for crown root development, and its mutation may result in the accumulations of SA and JA and induce plant defense response. 

The *scr8* exhibits an abnormal crown root development under high temperature, which is involved in plant defense response. However, the regulating mechanism of plant defense response under high temperature and whether the mechanism in root is the same as that of shoot are not known. Therefore, identification of novel genetic loci that regulate crown root development and characterization of corresponding regulatory genes would extend our understanding of rice root development. Here, we reported *SCR8* gene as a new genetic locus controlling crown root development, in which it will be helpful to further explore the balance between root growth and defense response under the changing environment. 

## 4. Materials and Methods 

### 4.1. Plant Materials and Growth Conditions

The rice (*Oryza sativa*) *scr8* mutant was obtained from a natural variation of *indica* variety R1206 in the field. Genetic and phenotypic analyses were performed using the F1 and F2 generations of a cross between the *scr8* mutant and a *japonica* rice cultivar, WuYunGeng7(WYG7). The WYG7, R1206, SCR8, and F2 population were grown in the field at Hangzhou, Zhejiang Province, China, and Lingshui, Hainan Province, China, under natural growth conditions.

Hydroponic experiments and histological analysis were conducted using Yoshida’s culture solution [61]. Seeds were soaked in water at 37 °C for 2 days, followed by germination for 1 day at 37 °C. The most uniformly germinated seeds were sown in a 96-well plate. Two days later, the seedlings were cultured in Yoshida’s culture solution. All plants were grown in a chamber with a 12 h dark/12 h light cycle at 60% humidity. For the temperature sensitivity experiment, plants were cultivated at 20 ℃, 24 ℃, 28 ℃, and 32 ℃ from germination to 2 weeks old. For the temperature shift experiment, plants were grown at 20 °C or 32 °C to 7 days and then shifted to 32 °C or 20 °C. The root length was measured every day under the different temperature conditions [14]. 

### 4.2. Phenotypical Characterization and Histological Analysis

To investigate whether the *scr8* mutation affects rice yield, plant height, panicle length, tiller number, and grain number were determined for each of 15 independent plants. Grain length, grain width, and 1000-grain weight were measured by a ScanMaker i800 (MICROTEK, China). 

For paraffin sectioning, plant materials were fixed in 70% FAA overnight, and section analysis was performed, as described previously [62]. All sections were observed under a light microscope.

### 4.3. EdU Staining

EdU staining was performed using an EdU kit (C0071L, BeyoClick EdU-488; Beyotime), according to the manufacturer’s instructions and previous studies [6,27]. Plants at 3 days after germination (DAG) were immersed in 20 mM EdU solution for 2 h, fixed for 15 min in 4% paraformaldehyde in phosphate buffer (140 mM NaCl, 2.7 mM KCl, 10 mM Na2HPO4, 1.8 mM KH2PO4, 0.1%Tritonx-100, pH 7.2) at room temperature, and then in EdU detection cocktail. Root tips were observed under the GFP channel on a Carl Zeiss LSM 710 confocal microscope. 

### 4.4. Pathological Analysis

Rice plants were inoculated with *Xoo* strains PXO99A and Zhe173 by the leaf-clipping method at the heading stage, and lesion length was scored 15 days after inoculation [63]. At least 10 individual plants and three tillers of each plant were inoculated with one strain.

### 4.5. Determination of Free SA and JA by HPLC

For SA and JA measurements, seedlings were grown in culture solution for 7 days. The leaves and roots were separately collected and stored at –80 ℃. The different tissues were used for SA and JA measurements using a protocol reported previously. In short, 100 mg sample were frozen in liquid nitrogen immediately and ground to a fine powder. Homogenates were extract in 1 mL 90% methanol water solution at 4 °C overnight. The samples were centrifuged at 10,000 g for 10 min and supernatants were transferred to a new tube. The precipitates were re-extracted with 0.5 mL 90% methanol solution. The combined supernatants were vacuum-dried and dissolved with 20 μL trichloroacetic acid solution (1 mg/mL), mixed, and vibrated for 1 min. The mixture was extracted with 1 mL ethyl acetate and cyclohexane solution (V/V 1:1), and the upper organic phase was transferred to a new EP tube, followed by drying under nitrogen and dissolution with 0.5 mL mobile phase for the HPLC [64].

### 4.6. Map-Based Cloning 

To fine-map the mutated gene, *scr8* was crossed with WYG7, and 768 recessive individuals were generated from the F2 mapping population. For fine-gene mapping, new SSR, SNP, or InDel molecular markers were developed. Gene prediction within the 126.4 kb fine-mapped region on chromosome 8 was performed using the publicly available rice database Rice Genome Annotation Project (RGAP, http://rice.plantbiology.msu.edu accessed on 6 February 2013). Genomic DNA was extracted from the samples using the hexadecyltrimethylammonium bromide (CTAB) method [65]. The PCR mixture included 5 μL2 × PCR mix (Yeasen), 1 μL DNA template, 0.5 μL each of forward and reverse primers (10 μmol/L), and H2O to a final volume of 10 μL. The PCR amplification program was as follows: pre-denaturation at 94 °C for 4 min; denaturation at 94 °C for 30 s, annealing at 55–60 °C for 30 s (depending on the primers), extension at 72 °C for 30 s 40 cycles, and a final extension at 72 °C for 10 min. The PCR products were separated by electrophoresis on a 4% agarose gel, photographed, and the data stored in a gel imager for read. The primers used are listed in Table S1 in the Supplementary Materials.

### 4.7. RNA Extraction and qRT-PCR Analysis

Total RNA was isolated with TRIzol reagent (Takara) from the roots and leaves of different plants, and cDNA was synthesized with ReverTra Ace kit (Toyobo) and then diluted for qRT-PCR using the SYBR Green PCR Master Mix kit (Applied Biosystems) in ABI7900 (Applied Biosystems). The reaction mixtures contained 2 μL cDNA template, 10 μL2 × SYBR qPCR mix, 0.8 μL each of forward and reverse primers, and ddH2O to a final volume of 20 μL. The reaction program was 95 °C for 30 s, 95 °C for 5 s, 60 °C for 15 s, and 72 °C for 30 s for 40 cycles. Each reaction was performed in triplicate. The cycle threshold (Ct) method was used to calculate relative amounts of mRNA. Student’s *t*-test was used to analyze the significance of differences. The rice Os*Actin* (*Os04g0177600*) gene was used as the endogenous control, and three replicates were performed for all experiments. The primers used are listed in Table S1 in the Supplementary Materials.

### 4.8. RNA-Seq and Data Analysis

Plants were grown at 20 ℃ and 28 ℃, and the roots were harvested for total RNA extraction at the 7th day. Three biological replicates (each comprising roots from 10 individuals) were used for RNA-seq analysis. The RNA-seq libraries were constructed and sequenced using the Illumina HiSeq (Illumina company), and each sample obtained approximately 50,000,000 clean reads. The reads were mapped to Nipponbare reference genome according to the genome information (ensemble, IRGSP-1.0, INSDC Assembly, version 103.7) by Tophat. DEGs were identified by edgeR with FDR < 0.05 and |log2 (fold change) | > 1) [66]. GO enrichment analysis was implemented with agriGO (http://bioinfo.cau.edu.cn/agriGO accessed on 31 May 2021).

## Supplementary Materials

The following are available online at https://www.mdpi.com/article/10.3390/ijms22189868/s1.
